# Force-Induced Dynamical Properties of Multiple Cytoskeletal Filaments Are Distinct from that of Single Filaments

**DOI:** 10.1371/journal.pone.0114014

**Published:** 2014-12-22

**Authors:** Dipjyoti Das, Dibyendu Das, Ranjith Padinhateeri

**Affiliations:** 1 Department of Physics, Indian Institute of Technology Bombay, Mumbai 400076, India; 2 Department of Biosciences and Bioengineering, Indian Institute of Technology Bombay, Mumbai 400076, India; Semmelweis University, Hungary

## Abstract

How cytoskeletal filaments collectively undergo growth and shrinkage is an intriguing question. Collective properties of multiple bio-filaments (actin or microtubules) undergoing hydrolysis have not been studied extensively earlier within simple theoretical frameworks. In this paper, we study the collective dynamical properties of multiple filaments under force, and demonstrate the distinct properties of a multi-filament system in comparison to a single filament. Comparing stochastic simulation results with recent experimental data, we show that multi-filament collective catastrophes are *slower* than catastrophes of single filaments. Our study also shows further distinctions as follows: (i) force-dependence of the cap-size distribution of multiple filaments are quantitatively different from that of single filaments, (ii) the diffusion constant associated with the system length fluctuations is distinct for multiple filaments, and (iii) switching dynamics of multiple filaments between capped and uncapped states and the fluctuations therein are also distinct. We build a unified picture by establishing interconnections among all these collective phenomena. Additionally, we show that the collapse times during catastrophes can be sharp indicators of collective stall forces exceeding the additive contributions of single filaments.

## Introduction

A large number of biological functions such as mitosis, acrosomal processes and cell motility are controlled by cytoskeletal filaments, whose classic examples are microtubules and actin filaments within cells [1]. Cytoskeletal filaments have different molecular structures – the microtubule has a hollow cylindrical shape made of 13 proto-filaments, while actin has helical shape made of two proto-filaments [1], [2]. In spite of their structural differences, these filaments have similar kinetic processes. They polymerize by adding ATP/GTP-bound subunits. Inside a filament, ATP/GTP is irreversibly hydrolysed into ADP/GDP. The presence of this chemical switching (ATP/GTP hydrolysis) makes the growth dynamics *non-equilibrium* in nature, and produces two distinct subunit-states, namely ATP/GTP-bound and ADP/GDP-bound. These two subunit-states have very distinct depolymerization rates, and this heterogeneity produces interesting dynamics [3], [4].

In the literature, the dynamics of a single cytoskeletal filament has been studied extensively [1], [5]–[18]. Single microtubules are known to exhibit a phenomenon called “dynamic instability” where the filament grows with a certain velocity, and then collapses catastrophically generating a huge fluctuation in the filament lengths [4], [19]. It has been reported that single actin filaments and ParM filaments (homologue of actin in prokaryotes) also exhibit large length fluctuations, somewhat similar to microtubules [20], [21]. Given that these filaments bear load under various circumstances, scientists have also investigated how these filaments and their length fluctuations behave under force [22].

Extensive theoretical investigation, combined with experiments, have given us a good primary understanding of how these filaments behave at the single filament level. Early phenomenological models tried to capture the filament dynamics by a two-state model [6] with stochastic transitions between growing and shrinking length-states. Later models incorporated detailed chemical processes such as binding and unbinding of monomers, and hydrolysis, using experimentally measured rates [12]–[14], [16]. All these studies revealed that the chemical switching (hydrolysis) is crucial to explain the experimentally observed feature of “dynamic instability” [4], [23] and similar large length fluctuations [12]. The reason behind this fluctuation phenomenon was found to be the formation of a ATP/GTP-cap at the filament-tip and the stochastic disappearance of it due to hydrolysis.

Although single-filament studies are helpful to understand the basic aspects of the dynamics, it is biologically more relevant to investigate a collective system of 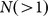 filaments. Even though scientists are starting to explore dynamics of multiple filaments under force experimentally [24], [25], the theoretical understanding of multi-filament dynamics and their fluctuations is minimal. Most of the existing models for multi-filaments neglect ATP/GTP hydrolysis and do not have any kind of chemical switching in their model [26]–[31]. Ignoring hydrolysis, for simple models of filaments with polymerization and depolymerization dynamics, exact analytical results for 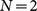 [26], [28], [29], and numerical results for 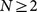 [27]–[30] have been obtained. Given that single-filament studies have already established the experimental importance of chemical switching [8], [11], [12], [32], it is crucial to have a multi-filament study where one takes into account the ATP/GTP hydrolysis in detail and investigate the dynamics. Also note that the irreversible process of hydrolysis makes the dynamics depart from equilibrium, and hence it needs careful consideration.

In the context of force generation, in a recent study, we have theoretically shown that ATP/GTP hydrolysis results in a new collective phenomenon [33]. For a bundle of 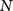 parallel filaments pushing against a wall, the collective stall force is *greater* than 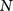 times the stall force of a single filament [33]. Earlier theories [27], [28] missed this effect as they neglected hydrolysis and studied equilibrium processes, which led to a notion that stall forces are additive for multiple filaments.

Apart from force generation, various fluctuations of the system-length during unbounded growth or “catastrophes” have been of great interest [12], [14], [16], [25]. Single-filament studies have described the length fluctuations by a measurable quantity, namely the diffusion constant [11], [12], [14], [26]. Recent theoretical studies of single actin filaments have shown that this diffusion constant has non-monotonic behavior as a function of monomer concentration [11], [12] – it has a peak near the critical concentration. It should be noted that such a peak would be absent without hydrolysis, which makes the filament switch between ATP/GTP “capped” and “uncapped” states [12]. Another aspect of length fluctuation is the catastrophe and rescue where the filament repeatedly grows and shrinks maintaining a constant average length [1]. Such stochastic length collapses recently have been observed for multiple microtubules in an experiment [25], and have been referred to as “collective catastrophes”.

A unified theoretical understanding of the above fluctuation properties (diffusion constant, catastrophes and cap dynamics) have not been provided in any earlier literature for multiple filaments under force, and undergoing hydrolysis. Zelinski and Kierfeld have theoretically studied the collective catastrophe using a phenomenological two state model [34]. However, none of the existing multifilament models take into account microscopic processes like polymerisation, ATP/GTP hydrolysis and depolymerisation of ATP/GTP- and ADP/GDP-bound subunits explicitly. Given that explicit dynamics at the subunit level is crucial in understanding the coupling between cap dynamics and length fluctuations, it is desirable to have a microscopic model that includes these features in detail.

Motivated by the above research background, in this paper we investigate the dynamics of multiple cytoskeletal filaments taking into account the kinetic events of polymerisation, depolymerisation, and ATP/GTP hydrolysis of subunits explicitly. The focus of the paper is to examine the collective properties that may emerge from the multifilament nature of the system, in the presence of force and non-equilibrium ATP/GTP hydrolysis. We show that collective behaviour of multi-filaments under force is qualitatively and quantitatively different from that of a single filament, and the ATP/GTP cap dynamics is crucial in understanding these phenomena. Examining the collapse during catastrophe, we show that the collapse time of a multifilament system is considerably higher than that of a single filament system; this indicates that the collective collapse of microtubules has a gradual nature as opposed to the sharp collapse of single microtubule. We find that this slow collapse of the multi-filament system is related to the enhanced stability of the ATP/GTP caps. We establish this by studying the cap-size statistics, and the switching dynamics of the system between capped and cap-less states. We find that the multifilament system has a non-zero cap, at any large force, while for a single filament cap vanishes at large forces. Finally, we show that these underlying features manifest in the macroscopic fluctuations of the system size and can be quantified as the experimentally measurable diffusion coefficient. Through this paper, we provide a unified picture by establishing connections between a number of collective properties of the multifilament system and the underlying kinetics of the AGP/GTP cap at the subunit level.

### Model

**Figure 1:**
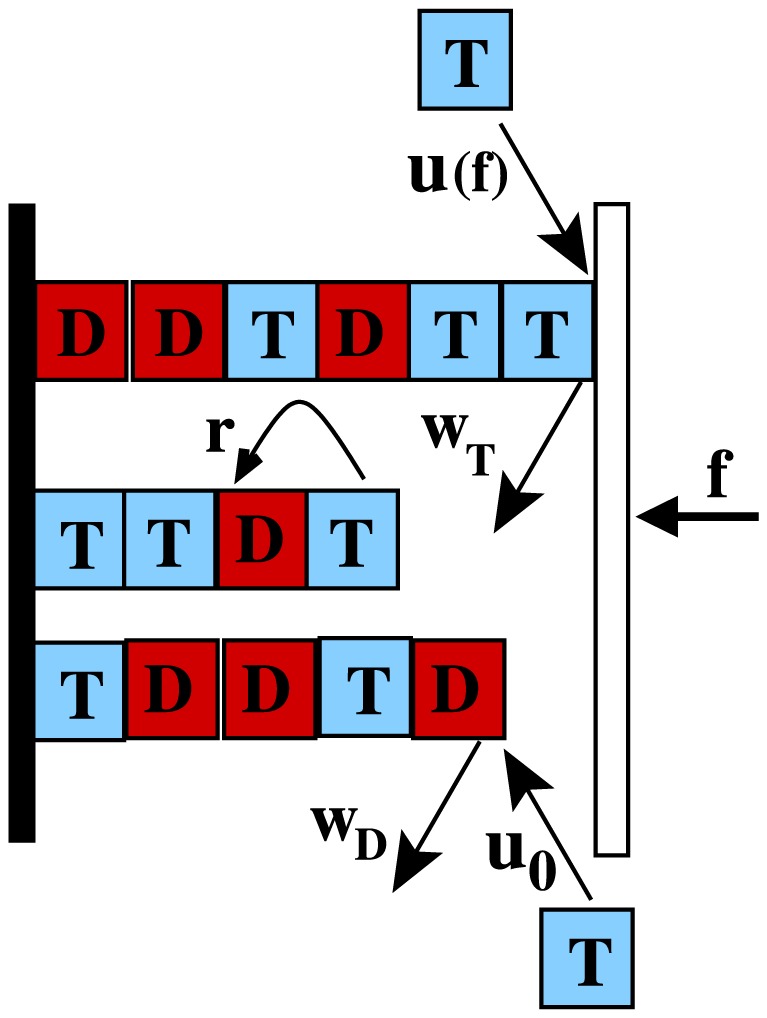
Schematic diagram of three-filament system with random hydrolysis, where the switching ATP/GTP → ADP/GDP occurs randomly at any ATP/GTP subunit. ATP/GTP and ADP/GDP subunits are shown as letters ‘T’ (blue) and ‘D’ (red) respectively. The left wall is fixed, while the right wall is movable with an externally applied force *f* pushing against it. Various possible events (as described in the text) are shown with arrows and corresponding rates.

We study a model of multiple cytoskeletal filaments as shown in Fig. 1, where 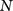 parallel and rigid filaments (actin filaments or microtubules), each composed of subunits of length *d*, are growing against a wall under a constant opposing force *f*. This model is a generalisation of the one-filament model studied in [16], to a multi-filament case. Note that this one-filament model was shown to have features similar to many experiments [22], [35] on single actin and microtubule, including catastrophe frequencies and length fluctuations [11], [12], [16]. In the literature, different groups have studied various models starting from highly coarse-grained two state models [6]–[8], [34] to vastly detailed model for single microtubules, taking into account its multi-protofilament nature [9], [10], [18], [36]. In the degree of coarse graining, our model falls somewhere in the middle – unlike the two-state models, our model takes into account microscopic processes of polymerisation, depolymerisation and hydrolysis at the level of subunits, explicitly. However, we do moderate coarse-graining such that a multi-protofilament system is represented as a single filament with appropriate subunit lengths – this middle level of coarse-graining has the advantage that it does not leave out the crucial microscopic kinetic events/features (hydrolysis, cap etc) and, at the same time, has only minimal number of parameters. The effective subunit lengths are taken to be 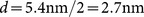 for actin filaments, and 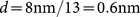 for microtubule, which accounts for the actual multi-protofilament nature of the biofilaments [13], [14], [16], [34] in a coarse-grained way. Explicitly, each filament grows by polymerisation of free ATP/GTP-bound subunits in a force-dependent manner. Filament tips away from the wall polymerise with a rate 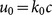. Here, 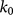 is the intrinsic polymerization rate-constant and 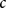 is the free ATP/GTP subunit concentration. The polymerization rate for the leading filament, which is in contact with the wall, is reduced due to the applied force *f* – according to the Kramer's theory, the rate becomes 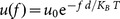 [27], [28]. Inside each filament, any ATP/GTP-bound subunit may get hydrolysed to a ADP/GDP-bound subunit randomly at any location with a rate *r*. This mechanism of hydrolysis is known as *random hydrolysis* [12], [16], [37]. In the literature other mechanisms of hydrolysis have also been proposed, namely *sequential* hydrolysis [11], [13] and *mixed cooperative* hydrolysis [8], [38], [39]. In this paper, we consider the random hydrolysis model, as it is thought to be closer to the biological reality [40]. Note that the chemical switching (ATP/GTP → ADP/GDP) is *non-equilibrium* in nature, as it is irreversible. For actin, the subunits also exist in an intermediate state bound to ADP-P_i_ [12], [40], [41] i.e. actin hydrolysis involves two steps in reality (ATP→ADP-P_i_→ADP). There are also reports indicating the relevance of a similar GDP-P_i_ intermediate state for microtubules [42], [43]. But we would consider only the dominant rate limiting step of P_i_ release (neglecting the ADP-P_i_ and GDP-P_i_ states), as was done in earlier literature [11], [13], [17]. Finally, the ATP/GTP-bound and ADP/GDP-bound subunit may dissociate from the tip of a filament with distinct force-independent depolymerization rates 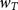 and 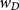 respectively. Although the depolymerization rates are assumed to be constants here, they can also depend on force – such a scenario has been briefly discussed towards the end of the paper. The continuous ATP/GTP stretch at the tip of a filament is called a “cap” – for example, in Fig. 1, the top filament has a cap whose size is two subunits. Note that the immovable left wall (see Fig. 1) acts as a reflecting boundary – this is equivalent to a filament growing from a fixed seed on the wall, where the filament can polymerise back once its length reduces to zero. We do kinetic Monte-Carlo simulations [44] of the above model using known rates for cytoskeletal filaments (see Table 1) to calculate various dynamical quantities, and the results are given below.

**Table 1:** Rates for Actin [1], [3] and Microtubules (MT) [1], [4], [19].

	*k*_0_ (*µM* ^−1^ *s* ^−1^)	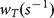	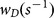	*r* (*s* ^−1^)
Actin	11.6	1.4	7.2	0.003
MT	3.2	24	290	0.2

## Results

### Collapse times reveal novel collective behaviour during catastrophe under force

In this section, we study the collective collapse of 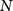 filaments during catastrophes. We simulate an 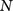 filament system growing against a wall under external force *f*, as discussed above. When the external force is larger than the “stall force” (maximum force) of the N-filament system (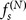), the filaments will not grow on an average – the system will be in a bounded phase of growth (see S1 Figure in S1 File).

**Figure 2:**
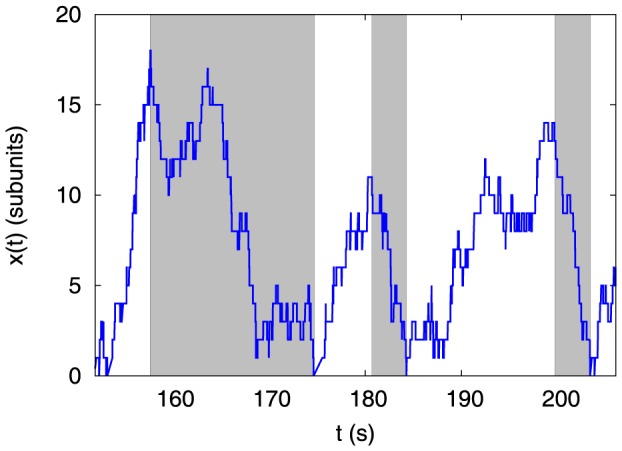
A time trace of the wall position *x*(*t*) for two microtubules (*N*  =  2) in the bounded phase, showing “collective catastrophe”, at a concentration *c*  =  100*µ*M 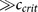, and at a force *f*  =  36.8 pN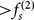 (*c_crit_*  =  8.67*µ*M, and 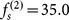
pN in this case). Other parameters are taken from Table 1. The regions shaded grey correspond to the catastrophes, and provide the collapse time intervals whose average is *T*_coll_.

First of all, our model shows collective catastrophes of multiple filaments in the bounded phase, similar to a recent experiment [25]. A typical time trace of the wall position (or equivalently system-length) is given in Fig. 2, where a system of two microtubules repeatedly grows from a length of zero to a maximum value and then shrinks back to zero. This stochastic collapses of the system-length from a local maximum to zero, would be referred to as “catastrophes”. Note that long stretches of shrinkage, not always going to zero length, also have been termed as catastrophes [1]. But such a definition would require an arbitrary minimum cut off length to count events of catastrophe. For simplicity, we consider this minimum length to be zero. To quantify and systematically investigate the catastrophe events, we define a measurable quantity called collapse time below: following Fig. 2, we define a “peak” as the furthest wall position between two successive zero values of the system-length (*x*). Then we define the collapse time (*T*_coll_) as the time it takes, on an average, to collapse from a peak to the next zero of the system-length (see the regions shaded grey in Fig. 2). Below stall force, where the system would be in a unbounded growing phase (see Appendix A in S1 File), the *T*_coll_, according to our definition, would be infinite as the trajectories of the system-length would no longer collapse to zero (on an average). In other words, *T*_coll_ is expected to diverge for 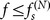. On the other hand, *T*_coll_ should be finite in the bounded phase (see Fig. 2) as there are frequent catastrophes. Thus, the finiteness of *T*_coll_ values is a quantitative indicator of the existence of catastrophes.

**Figure 3:**
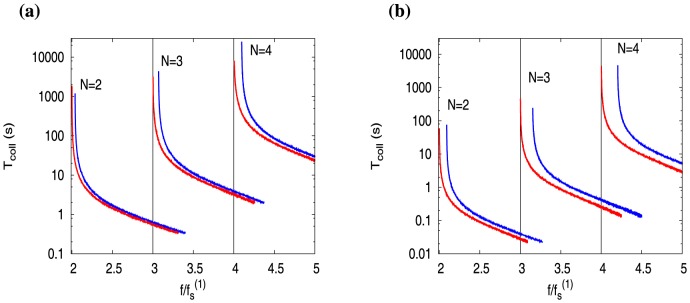
Average collapse times *T*_coll_ as a function of scaled force 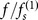 with increasing number of filaments (***N***), for (a) actin filaments and (b) microtubules. Blue and red curves are with hydrolysis (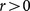) and without hydrolysis (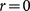) respectively. The curves are plotted by scaling the force-axis with corresponding single-filament stall forces. For 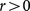, the numerically obtained values of single-filament stall forces are 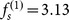 pN for actin, and 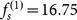 pN for microtubule. While, for 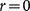, the corresponding single-filament stall forces are obtained from the formula 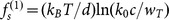 (see [27]) – these are 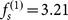 pN for actin, and 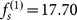 pN for microtubule. Parameters are taken from Table 1. The ATP/GTP concentrations are 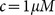 for actin, and 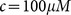 for microtubules.

In Fig. 3, we plot *T*_coll_ as a function of scaled force 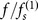, for multiple actin filaments (Fig. 3a, blue curves) and microtubules (Fig. 3b, blue curves). As expected, at large forces, the values of *T*_coll_ are finite, corresponding to the bounded phase. However, they diverge at specific force values which are nothing but the collective *stall forces* of N filaments 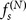. Interestingly, we see that 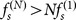. This collective phenomenon of excess stall force generation (as opposed to 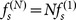) was recently discovered by us [33]; we had obtained 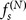 by computing the force at which 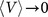 (see [33]). Note that here we are estimating 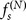 from the 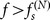 regime (bounded growth phase), while in [33], the approach was from the 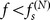 regime (unbounded growth phase) – see S1 Table in S1 File (Appendix B) for a comparison. It is important to stress that if hydrolysis is ignored, i.e. for the hydrolysis rate *r* = 0, one obtains the red curves in Fig. 3 — they show 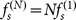, a widely believed result, but nevertheless actually untrue in reality. We have also observed that the inverse of the collapse time (equivalent to rate), for single filament at zero force, decreases with increasing tubulin concentration – this trend is similar to many of the single filament experiments [35], [45].

As *T*_coll_ is a nice quantitative measure of catastrophes, we would like to use it to address two questions: (a) is nature of the catastrophe of multiple filaments (collective catastrophe) different from that of a single filament? (b) is there any difference between zero-force catastrophe and force-dependent collective catastrophe? Before proceeding to answer these two questions, we note that two external factors can control the catastrophe – force and concentration of subunits (see Appendix A in S1 File). In the absence of any force, all filaments are independent of each other, and therefore the average behaviour of N filaments is exactly the same as that of a single filament. However, in the presence of force, the filaments interact via the movable wall. Due to the applied force, the growth rate of a filament, which is otherwise 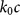, reduces instantaneously to 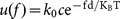, the moment it touches the wall. By this mechanism the trailing filaments get affected by the spatial location of the leading filaments. This implicit interaction among filaments for 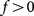, can potentially lead to new collective phenomena for multi-filament systems, as we would show soon.

**Figure 4:**
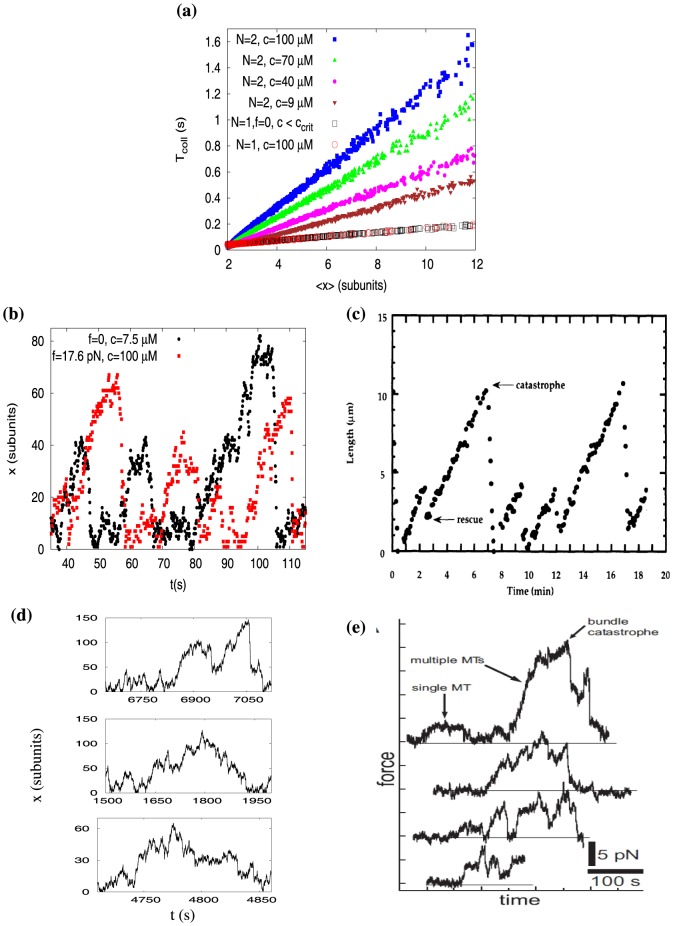
(a) Plot of *T*_coll_ versus 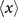 obtained in the bounded phase, for microtubules. Black squares are for 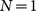, 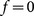, 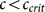, and red circles are for 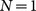, 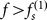, 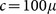
M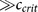 (for microtubule parameters, 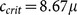
M). While, for 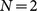, 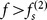, values of *T*_coll_ are obtained at four different concentrations (all greater than 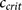) 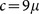
M (brown down triangles), 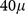
M (magenta bullets), 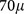
M (green up triangles), and 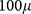
M (blue filled squares). (b) Two simulated time-traces of the wall-position 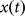 for a single microtubule, for parameters: (i) 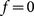, 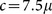
M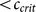 (black dots); (ii) 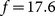 pN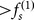, 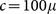
M (red squares). (c) An experimental trajectory (reproduced from [46]) of length versus time for a single microtubule under zero force (Copyright (1994) by the American Physical Society). (d) Three different time-traces of the wall-position for two microtubules (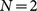) from our simulations. Parameters are: 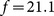 pN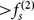, 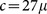
M. (e) Experimental trajectories (reproduced from [25]) of a bundle of multiple microtubules under harmonic force – this force, shown in y-axis, is proportional to microtubules' extensions (*x*) (Copyright (2008) by National Academy of Sciences, U.S.A).

Noting these points, we proceed to compare the catastrophes for the following three cases: (i) 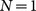, 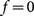, 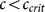, (ii) 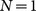, 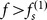, 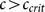 and (iii) 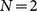, 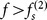, 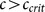. Since the parameter regimes of the three different cases are very distinct, we present a scatter plot (see Fig. 4a) between the collapse time (*T*_coll_) and the average length of the leading filament (or the mean wall position). Firstly we see that for a single filament (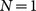), the *T*_coll_ data for the case (i) (by varying *c*), and for the case (ii) (by varying *f*), completely collapse on to each other (see bottom curves with the symbols of black squares and red circles in Fig. 4a). This means that the average collapse times of a single filament with or without force are similar. But, the situation is strikingly different for 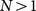 in presence of a force. For 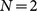 microtubules (case (iii)), we calculated the values of *T*_coll_ at four different concentration values greater than 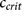 (blue, green, magenta and brown symbols in Fig. 4a) by varying forces 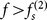. We clearly see that the values of *T*_coll_ are much higher compared to those of 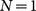, for the same given average length. This implies that, during catastrophes of 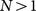 filaments under force, the system-length collapses more *slowly*, than a single filament.

In Fig. 4b, we show two trajectories of a single microtubule from our simulation for the cases (i) and (ii). We see sharp length collapses for 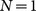 – the trajectories without force (case (i)) and with force (case (ii)) both looks similar. This should be compared with the experimentally obtained trajectory of a single microtubule under zero force [46], reproduced in Fig. 4c – the simulated trajectory in Fig. 4b (black dots) and the experimental trajectory in Fig. 4c both have sharp catastrophes. On the contrary, the simulated trajectories for 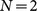 (case (iii)) show comparatively much gradual catastrophes – see Fig. 4d. The experimentally obtained trajectories [25] of multiple microtubules (Fig. 4e) also show similar behavior. Although the experiment [25] corresponding to Fig. 4e is done under harmonic force (unlike our theoretical model with a constant force), the comparisons of our simulation with the experiments provide an interesting insight. The catastrophes in multi-filament system seem to be *slower* than that of a single filament.

**Figure 5:**
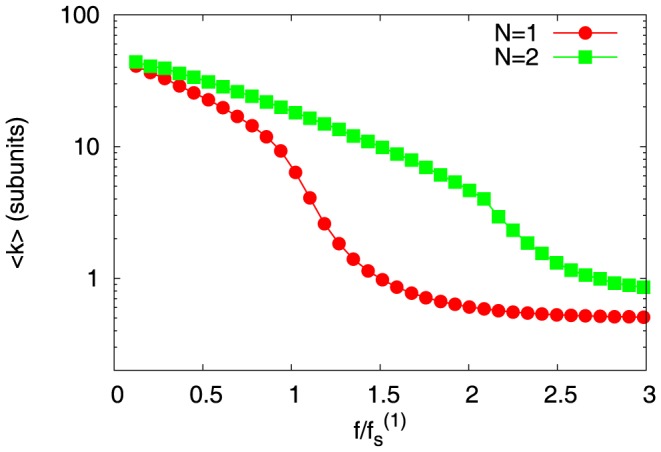
Average cap size 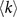 as a function of scaled force 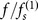 for microtubules, and for filament numbers 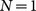 (red), and 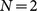 (green). The system is in the bounded phase for forces greater than the stall forces. The GTP concentration is 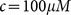, and other parameters are specified in Table 1. The Y-axis is in log scale.

Above observations clearly indicate that, the system of multiple filaments under force seem to be more “stable” in comparison to a single filament during their catastrophes in the bounded phase. By “stability” we mean that multiple filaments resist the opposing force more effectively and thus collapse more slowly compared to 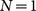. Sudden collapse, during catastrophe, is typically associated with the disappearance of ATP/GTP cap and exposure of ADP/GDP bulk, while the stability is associated with the presence of the ATP/GTP cap. This raises an obvious question: Do slow collapses during collective catastrophe, exhibited by the multi-filament system, have something to do with ATP/GTP cap stability? To get a preliminary understanding, we calculated the average cap sizes 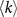 as a function of force, for 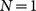 and 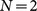 in the bounded phase — this is shown in Fig. 5. This figure clearly shows that average cap sizes of a two-filament system is greater than that of a one-filament system. This points to a new cap structure for collective (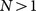) dynamics. In the next section, we examine these collective effects on cap size statistics and cap dynamics in detail.

### Multiple filaments under force show distinct cap-size statistics

In this section, we study the statistics of ATP/GTP cap-sizes with the aim of understanding how it renders stability to a multi-filament system and slows down the catastrophe. Since our goal is to understand the steady-state properties of the caps during catastrophe, we start with very long filaments. By studying the shrinkage of such filaments we can examine the collective behaviour of their caps, without any boundary effect that may arise from the rigid wall at zero length.

**Figure 6:**
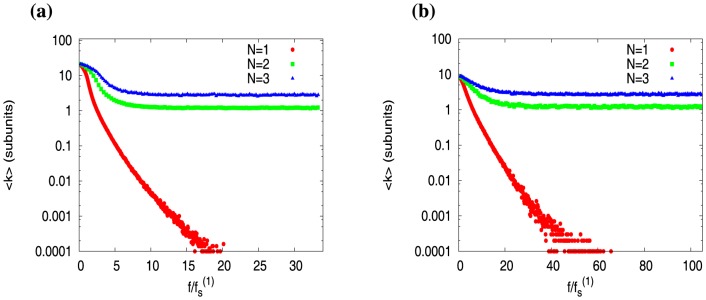
Average cap size 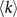 as a function of 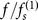 for (a) actin filaments and (b) microtubules, and for filament numbers 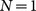 (red), 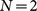 (green) and 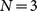 (blue). The concentrations are 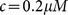 for actin, and 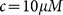 for microtubule. Y-axes are in log scale. Note that the single filament stall forces are 0.68 pN for actin and 0.97 pN for microtubule.

In Fig. 6, we plot 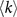 against the scaled force 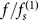, for actin filaments (Fig. 6a) and microtubules (Fig. 6b). Note that this figure is the counterpart of Fig. 5, that was studied for short filaments with possible boundary effects (see previous section). In Fig. 6, when 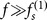, we see that mean cap-length 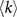, for single filament, rapidly decays to zero (see red curves in Fig. 6). There is a distinction between actin versus microtubule though – the force range over which cap is present is larger for microtubule than actin. However for 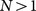, 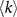 does not vanish at all — rather, it first reduces and then *saturates* (or stabilizes) to a finite value of 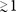 subunits, at forces 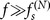 (see green curves for 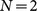, and blue curves for 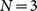 in Fig. 6). These results reaffirm our observation in the last section that the multifilament system does show a distinct cap structure – while average cap length of a single filament is vanishingly small, the multifilament system always has a non-vanishing larger cap. Does this also reflect in the full cap size distribution?

**Figure 7:**
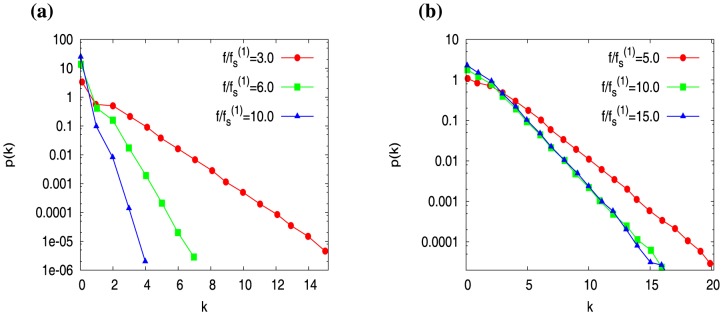
Distributions of cap sizes, 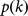 at different forces for (a) single actin (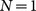), and (b) two actin filaments (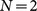), for a concentration 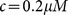. Y-axes are in log scale.

In Fig. 7a, we plot the cap-size distributions 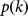 for a single actin filament at three different force values. We clearly see that the cap-size distributions for 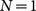 have decreasing widths with increasing force. This trend, if continued, would lead to a vanishing cap as 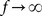. However, we see a different picture for 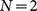 filaments (Fig. 7b) – the distribution 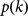 saturates with increasing force, implying a non-vanishing cap for multiple filaments.

This phenomenon can be understood by noting the following: for a multi-filament system (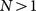), only the leading filament “feels” the force; the trailing filaments have force-independent rates. Therefore the trailing filaments have much higher polymerisation rates (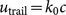) compared to the leading one (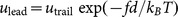), and hence they acquire ATP/GTP subunits at the tip. In other words, the trailing filaments “catch up” with the leading filaments by polymerising ATP/GTP subunits. Thus, in a multifilament system there exists a finite cap, always, even at large forces, unlike the single filament.

In summary, we have discovered a collective phenomenon that the cap-sizes stabilize with increasing force for multiple filaments, unlike a single filament. This in turn imparts enhanced stability to multiple filaments during their catastrophes (as discussed in the last section). Note that it is possible to experimentally visualize the GTP-cap by using a suitable conformational antibody that specifically recognizes GTP-bound tubulin in microtubules [47]. Such techniques may be used to experimentally validate our predictions for cap sizes (which are 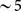 subunits for 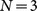, see Fig. 6). Another experimental way to observe the consequence of above phenomenon may be the measurement of collapse time *T*_coll_ (as discussed in the previous section). Alternatively, one may investigate experimentally the macroscopic length fluctuations of multi-filament system, which is quantified in the diffusion constant [20]. Do the length fluctuations bear any quantitative signature of the collective effect of cap-size stabilization? We shall investigate this question in the next section.

### Collective behaviour in diffusion coefficient for length fluctuations of 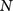 filaments

In this section we investigate fluctuations of the overall system-length (wall position) of an N-filament system under force, and examine plausible collective effects. The length fluctuations can be characterised by the diffusion constant for the wall position:
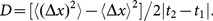
(1)


Here 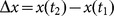 is the difference between two distinct instantaneous wall positions, measured at times 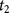 and 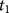 respectively. We calculate 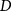 at the steady state (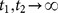) where it is independent of time and for the full range of forces below and above 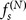.

In the literature, different groups have examined the diffusion constant for a single actin filament (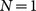) as a function of ATP-bound monomer concentration (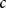) at zero force [11], [12]. It was found that 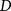 has a pronounced peak near critical concentration (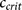). This non-monotonic behaviour of 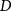 was attributed to transitions between capped state and uncapped states, as a result of ATP hydrolysis. Without hydrolysis, the filament has no such transition between two states and hence 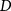 is monotonic. However, the behaviour of 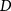 for a multifilament system, under force, is currently unknown.

**Figure 8:**
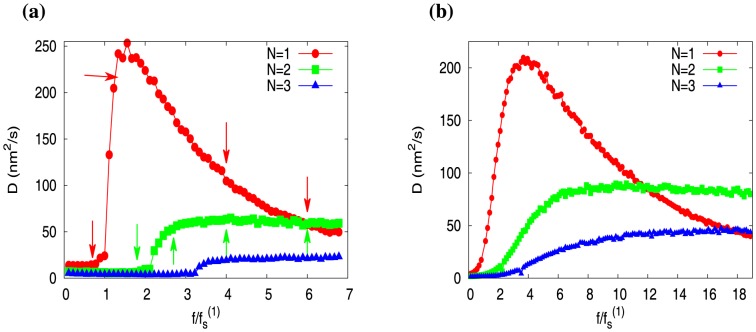
The diffusion constant 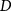 of the wall position as a function of scaled force 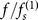 for (a) actin filaments and (b) microtubules, with filament number 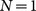 (red), 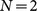 (green) and 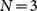 (blue). Concentrations are 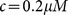 for actin and 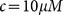 for microtubule (for other parameters see Table 1). In (a), the arrows correspond to the force values at which we shall investigate the cap dynamics of the filaments in the next section (see Fig. 9).

We now present our results for diffusion coefficient 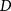 in Fig. 8, as a function of scaled force 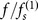, both for actin filaments (Fig. 8a) and microtubules (Fig. 8b). For one filament (red curves in Figs. 8a and 8b), we see that 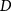 rises up near the stall force 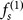 and goes to zero as 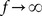. Like refs. [11], [12], we note that the non-monotonic behavior of 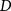 is an effect of hydrolysis — we have checked that this is absent for hydrolysis rate 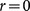. What is striking is that for 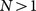, 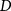 curves have a distinct feature compared to 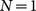 (see green curves for 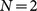 and blue curves for 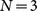 in Figs. 8a and 8b). For 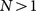, we see that 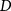 curves rise up near the corresponding stall forces 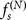, but they do not decay to zero at large forces like the 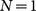 case — in fact, they *saturate* with force. As a result, the length fluctuations of a multifilament system is larger than that of a single filament system as 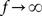.

The collective effect is reminiscent of the stabilization of caps with force for 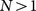 seen in the previous section. But, how exactly the microscopic dynamics of the caps contribute to the macroscopic length fluctuation? This may be understood by examining the transitions between “capped” and “uncapped” states of the filaments. In the next section we proceed to study these transitions as a function of applied force.

### System length fluctuations are related to fluctuations in switching between capped and uncapped states

In this section we demonstrate how transitions between capped and uncapped states of the filaments play a crucial role in the fluctuations of the wall position. To describe the instantaneous state of the tip of a single filament (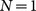), we first define the following stochastic variable:
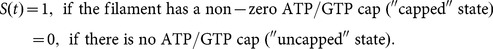
(2)


Above definition can be extended to multiple filaments. For 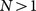, we define 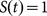 or 0 depending on whether the “leading” filament is capped or uncapped. Note that state of the leading filament is connected to the dynamics of the wall.

**Figure 9:**
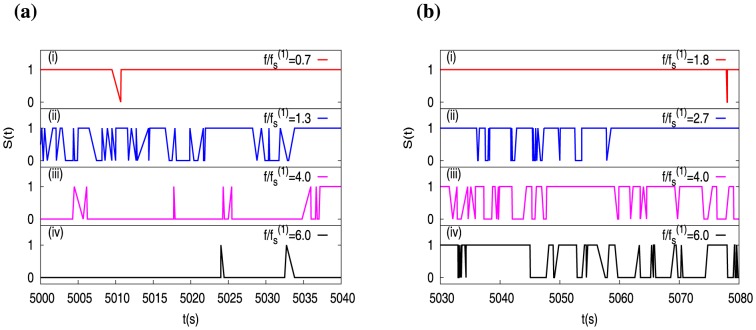
Few time traces of 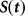 of the leader for (a) 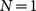 and (b) 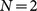 actin filaments, at a concentration 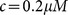 and at different values of scaled forces. At these forces, the corresponding values of wall-diffusion coefficient 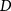 are shown by arrows in Fig. 8a (red arrows for 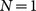 and green arrows for 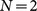).

In Fig. 9a we show the time traces of 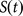 for a single actin filament at different force values – at these forces, the corresponding values of wall-diffusion constant *D* are shown by red arrows in Fig. 8a. We see that, at 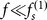 the filament is mostly in the capped state – 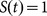 (mostly) in top panel (i) of Fig. 9a. When 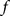 is just above 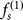, we see in panel (ii) of Fig. 9a, that there is a sudden increase in the number of switching events between capped and uncapped states. If 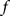 is increased further, the number of switching events decreases – see subsequent panels (iii) and (iv). So, the number of switching events first increases, and then decreases with force. Note that this behavior mimics the non-monotonic behavior of the wall-diffusion constant *D*, for 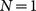 (see Fig. 8a). Moreover, the bottom panel (iv) of Fig. 9a, where 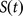 is mostly 0, signifies that the filament is capless (also see Fig. 6).

We now show the time traces of 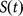 for two actin filaments in Fig. 9b, at different force values; see corresponding *D* values in Fig. 8b, marked by green arrows. Here we see a very distinct feature compared to the one-filament case — although the number of switching events increases first (see panels (i) and (ii) of Fig. 8b), it does not decrease with force, unlike the single filament case. In fact, the switching is present even at large forces – compare the histories in the last panels (iv) of Figs. 9a and 9b. Furthermore, in panels (iii) and (iv) of Fig. 9b the number of switching events are nearly the same, suggesting saturation with force. This saturation behavior for 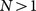, may be correlated with the saturation of the wall-diffusion constant *D* at large forces. To make this apparent correlations between *D* and the switching number fluctuations concrete, we now proceed to quantify the fluctuations in the number of switching events.

From the time traces of 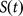, we first computed the number of switching events (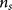) between the capped and uncapped states within a time window 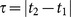. We then calculated the variance of 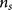 and found that the variance grows linearly with the size of time-window i.e. 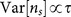. This enables us to construct a diffusion constant for the switching events as below:
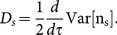
(3)


We compute 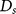 at large times, where it becomes independent of time.

**Figure 10:**
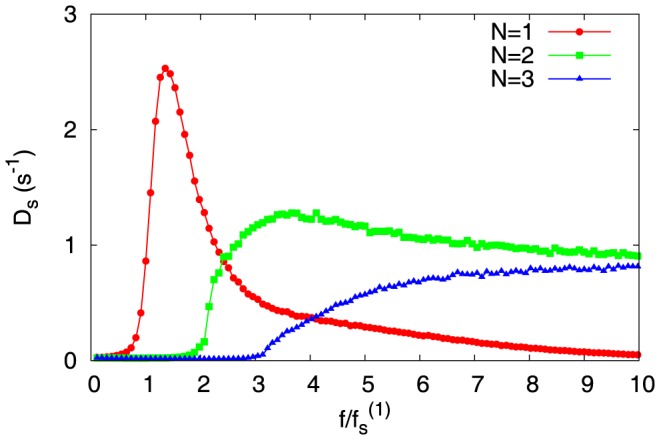
The diffusion constant 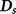 derived from the fluctuations of the switching events between the capped and uncapped states, is plotted against scaled force 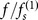. The data is for actin parameters (see Table 1) at concentration 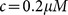, for filament-numbers 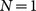 (red), 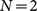 (green), and 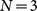 (blue).

In Fig. 10 we plot 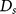 versus 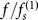 for actin parameters (see Table 1). Quite strikingly, we see that behavior of 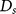 is very similar to the behavior of wall-diffusion constant *D* (see Fig. 8a). Just like the wall-diffusion constant, at large forces, 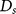 goes to zero for 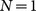, and it saturates for 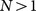. This clearly demonstrates that the wall-position fluctuations (quantified by *D*) are closely tied to the fluctuations of the switching events (quantified by 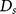) between the capped and uncapped states.

## Discussion and Conclusion

The current understanding of dynamical properties and fluctuations of cytoskeletal filaments, with hydrolysis, is mostly based on studies of single filaments [5], [8], [11]–[14], [16]. Recent experiments by Laan et al [25] and subsequent theory papers have started exploring various aspects of multiple filament systems under force [33], [34]. It has been proposed in [34] that the catastrophe rate should increase rapidly with force, in order to observe collective catastrophe and rescue oscillations. We note that the random hydrolysis model that we are using already has catastrophe rates that obey the criterion [16] and are comparable to the experimental results [22], [35]. Moreover, the microscopic nature of our model can provide clearer understanding of mechanisms leading to catastrophe, length fluctuations and cap dynamics of a mutifilament system. In this paper, using multiple filaments under force, taking into account polymerisation, ATP/GTP hydrolysis and depolymerisation of T- and D-bound subunits, we systematically investigated and clarified a number of aspects related to the dynamics and fluctuations of the system. Specifically, we showed that the fluctuations during collective catastrophes, the fluctuations of the ATP/GTP cap sizes, and the system length fluctuations, all are closely tied to each other. The unified picture emerging from these studies show that the collective behaviour of multiple filaments are quantitatively distinct from that of a single filament under similar conditions. For example, multifilament systems are more stable during catastrophe, when compared to a single filament system. Thus, our study suggests that it would be inaccurate to generalise the intuitions built on existing studies of single filaments to the more biologically relevant scenario of multiple filaments.

We quantified the fluctuations during catastrophes by the mean collapse time (*T*_coll_). We found that *T*_coll_ is systematically lower for a single filament compared to multiple filaments. This implies that the multi-filament system has an enhanced resistance against externally applied force. This will also clearly reflect in the experimentally measurable length versus time data, where, according to our prediction, the collective collapse will have a lower average negative slope, unlike the sharp collapse which is the hallmark of a single filament catastrophe (see Fig. 4c, and [46], [48]). Recent experiments on multiple microtubules under force clearly show this trend of slower collapse in their length versus time data (see Ref. [25] and Fig. 4e). This interesting feature, an understanding of which naturally emerges from our model, seems to be absent in time traces of wall positions obtained using other theoretical models in the literature (models in [25], [34]). Detailed study of our model under harmonic force needs to be done in future to achieve clearer understanding of such aspects in comparison to the works of [25], [34].

Exploring the ATP/GTP cap structure and statistics of individual filaments in the multifilament system, we found those to be highly stable at large forces. This enhanced stability of the caps (for 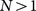) imparts stability to a multi-filament system, which is responsible for their slow collapse. Moreover, the stability of the caps is also reflected in the macroscopic length fluctuations of *N* filaments, which we quantified by a diffusion constant (*D*). We find that, at large forces, the value of *D* (for 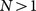) saturates – this experimentally observable effect owes its origin to the number fluctuations of the switching events between the capped and uncapped states (quantified by 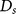). The similarity of the curves of *D* and 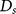 (versus force) demonstrates this. (see Figs. 8a and 10). In single microtubule dynamics, presence of GTP-bound subunits in the bulk is associated with rescue [47]. In multifilament systems one would expect enhanced rescues, at smaller forces closer to the stall force, as the lagging filaments can easily acquire GTP-bound subunits.

Although challenging, the caps may be directly observable experimentally using novel techniques [47]. Other quantities like the collapse time *T*_coll_ and the diffusion constant *D* can also be measured in a laboratory. Note that our definition of *T*_coll_ and *D* rely on just the time traces of the system length, which can be obtained easily in well designed experiments. It is worth mentioning that *T*_coll_ may be used to determine the stall force of a multifilament system and its deviation from the additive law (i.e. 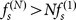), as predicted in our earlier work [33], can be verified.

Let us discuss the implications of relaxing some of the assumptions underlying our model. (i) One assumption was that of the force independence of the depolymerisation rates. In general, one may expect rates as follows: 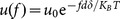, 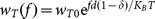, and 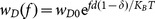, where the parameter 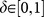 is known as the force distribution factor [28], [49]. Interestingly, experiments on microtubule [49] estimated 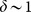, i.e. depolymerisation rate is force-independent just as we considered in this paper. However for actin, 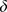 is not known experimentally. In S2 Figure of S1 File (Appendix C), we show the average cap sizes and diffusion constants for actin for 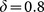 – the results are unchanged qualitatively. A detailed study of the 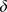 dependence of different dynamical properties studied in this paper would be undertaken in future. (ii) We considered the bundle of filaments without any lateral shift between the first monomers (seed). However, even if we introduce a lateral shift, the qualitative nature of our results are expected to remain the same. That is because the observed fluctuation properties in this paper are argued to be related to the switching between capped and uncapped states, which are unaffected by the lateral shift. (iii) Cytoskeletal filaments need not be perfectly rigid as we considered in this paper. A filament with finite stiffness can undergo buckling under external force. This may be avoided if one keeps the filaments short, below a critical length [48]. We estimate the critical length for buckling to be 4–17* µm* (for *c* = 10–100 µM) for microtubule, and 0.5–3* µm* (for *c* = 0.15–1* µM*) for actin, at their respective stall forces. So buckling can be prevented by choosing the lengths well below the critical lengths of the filament, as done in the experiment of Laan et al [25]. Note that, thermal fluctuations may alter the critical lengths for buckling as discussed by Emanuel et al [50]. Even in the absence of overall buckling, bending fluctuations due to thermal forces may generate gaps that are large enough to accommodate monomers leading to a change in polymerisation rates. However, some calculations show [51] that the effects of thermal fluctuations on polymerization rates would be negligible in the large force limit – a regime where we do most of our calculations. A detailed study of the role of thermal fluctuations is beyond the scope of the current work and may be performed in the future.

We would like to conclude by pointing out that dynamics of cytoskeletal filaments under any situation providing a scope for cooperativity (e.g, a boundary wall held by a force, as in our case) may produce surprises for multi filaments. Alternatively filaments may interact with each other via explicit lateral interactions (which was not considered in this paper) – this may also produce interesting dynamical effects. Understanding of such situations should start with case studies of at least two filaments. Any conclusion based on single filament study, in such cases, would be misleading.

## Supporting Information

S1 FileIncludes supporting figures and table. **S1 Figure.** (a) Phase diagram of 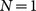 microtubule in the force (f)-concentration (c) plane. The curve of mean wall-velocity 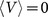 demarcates between two phases, namely the bounded and unbounded growth phases. (b) and (c): Typical time traces of the wall position in the bounded phase. The trajectory of (b) shows that the system length (wall position) 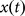 first shrinks rapidly with a negative velocity, but ultimately it fluctuates around a constant mean value — the later part is zoomed in (c), which shows catastrophes of the filament. (d) A typical trajectory of the system length in the unbounded growth phase, where 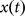 grows in time with a positive velocity. Parameters are specified in Table 1 and inside the figure panels. **S2 Figure.** (a) Average cap size 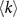, (b) the diffusion constant 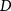 for the system-length fluctuations, and (c) the diffusion constant 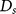 for the fluctuations of switching events between capped and uncapped states — these are plotted against the scaled force 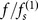. All data are for actin parameters (see Table 1) with a concentration 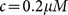 and for 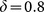. **S1 Table.** Comparison of values of stall forces obtained numerically by monitoring the limits 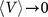, and 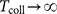. ATP/GTP Concentrations are taken to be 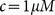 for actin and 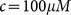 for microtubule (for other parameters see Table 1).(PDF)
