# Supplementary material for: Force-Induced Dynamical Properties of Multiple Cytoskeletal Filaments Are Distinct from that of Single Filaments
Source: PLoS One. 2014 Dec 22;9(12):e114014. doi: 10.1371/journal.pone.0114014 (PMC4273989; doi:10.1371/journal.pone.0114014)
Supplement: S1 File — Includes supporting figures and table. S1 Figure. (a) Phase diagram of microtubule in the force (f)-concentration (c) plane. The curve of mean wall-velocity demarcates between two phases, namely the bounded and unbounded growth phases. (b) and (c): Typical time traces of the wall position in the bounded phase. The trajectory of (b) shows that the system length (wall position) first shrinks rapidly with a negative velocity, but ultimately it fluctuates around a constant mean value — the later part is zoomed in (c), which shows catastrophes of the filament. (d) A typical trajectory of the system length in the unbounded growth phase, where grows in time with a positive velocity. Parameters are specified in Table 1 and inside the figure panels. S2 Figure. (a) Average cap size , (b) the diffusion constant for the system-length fluctuations, and (c) the diffusion constant for the fluctuations of switching events between capped and uncapped states — these are plotted against the scaled force . All data are for actin parameters (see Table 1) with a concentration and for . S1 Table. Comparison of values of stall forces obtained numerically by monitoring the limits , and . ATP/GTP Concentrations are taken to be for actin and for microtubule (for other parameters see Table 1). (PDF) [file pone.0114014.s001.pdf]

Appendix A: Different phases of growth and shrinkage

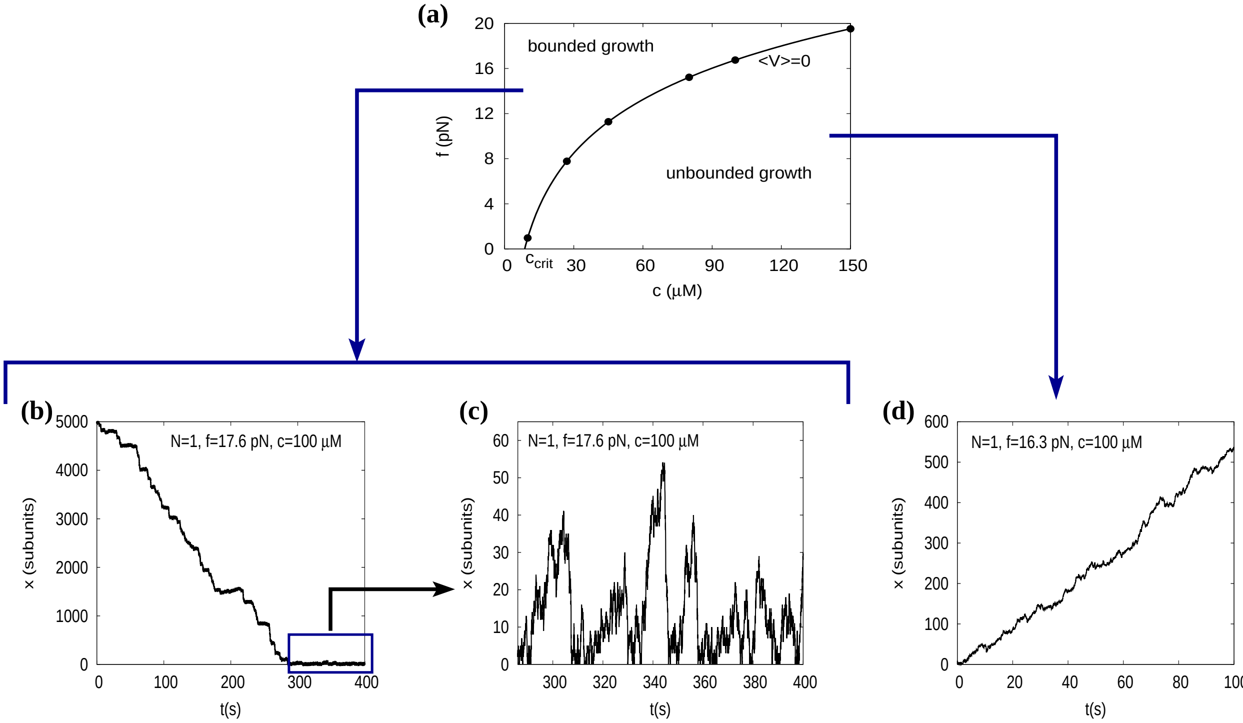

Fig. S1. (a) Phase diagram of $N = 1$ microtubule in the force (f)-concentration (c) plane. The curve of mean wall-velocity $\langle V \rangle = 0$ demarcates between two phases, namely the bounded and unbounded growth phases. (b) and (c): Typical time traces of the wall position in the bounded phase. The trajectory of (b) shows that the system length (wall position) $x(t)$ first shrinks rapidly with a negative velocity, but ultimately it fluctuates around a constant mean value — the later part is zoomed in (c), which shows catastrophes of the filament. (d) A typical trajectory of the system length in the unbounded growth phase, where $x(t)$ grows in time with a positive velocity. Parameters are specified in Table 1 and inside the figure panels.

Depending on the values of the applied force f and concentration c, there are two dynamical phases of a N-filament system — (i) the bounded growth phase, and (ii) the unbounded growth phase $[1, 2]$. The phase diagram for $N = 1$ microtubule is shown in the $f - c$ plane, in Fig. S1a. The $\langle V \rangle = 0$ curve marks the phase-boundary (where, $\langle V \rangle$ is the mean wall velocity). It should be noted that, in the absence of force ($f = 0$), there exists a critical ATP/GTP concentration $c = c_{crit}$, at which $\langle V \rangle = 0$ i.e. the system is stalled (see Fig. S1a). In the presence of force ($f > 0$), and for a concentration $c > c_{crit}$, the system can only be stalled when we apply the "stall force", $f = f_s^{(N)}$ at which the average wall velocity $\langle V \rangle = 0$ $[3, 4]$.

For the parameter regime $c > c_{crit}$ with $f > f_s^{(N)}$, or for $c < c_{crit}$ with $f \geq 0$, the filament shrinks on an average with a negative velocity — see the trajectory of the wall position in Fig. S1b. During shrinkage, when the filament length becomes very short, the filament eventually encounters the immovable left wall, and then the length fluctuates around a constant mean value — see Fig. S1c. This is called the bounded phase. On the other hand, for the parameter regime $c > c_{crit}$ and $f < f_s^{(N)}$, the filament indefinitely grows on an average, with a positive velocity — this is the unbounded growth phase — see

a typical trajectory within this phase in Fig. S1d.

Appendix B: Comparison of stall forces obtained from the collapse time measurements, and from the force-velocity relations

In this appendix, we discuss two possible measurement procedures for the stall force. In theories, the stall force is usually measured from force-velocity relations by finding the force $f = f_s^{(N)}$, at which average wall velocity $\langle V(f) \rangle = 0$ [3,5,6]. But a fact is that $\langle V \rangle$ becomes very small for $f \to f_s^{(N)}$, with increasing N. As a result, for $N > 2$, the task of experimentally measuring the precise $f_s^{(N)}$ is challenging. In fact, from simulations we calculated that for $N = 3$ microtubules (or actin filaments), at a force $f = 3f_s^{(1)}$ and concentration $c = 100\mu M$ (or $c = 1\mu M$), the wall velocity is ~ 0.1 nm/s (or ~ 0.02 nm/s). Monitoring such slow motion, and finding the force for which the wall truly halts maybe difficult.

We have already shown in the main text that, if $f \to f_s^{(N)}$ from above, the average collapse time T_{coll} tends to diverge. This behavior of T_{coll} may be used to precisely determine the collective stall force $f_s^{(N)}$ of a system. This relies on approaching $f_s^{(N)}$ from above, as opposed to from below as in the case of $f - \langle V \rangle$ measurements. Note that such a measurement of T_{coll} should be easy experimentally, as one is dealing with large values, while monitoring vanishingly small values of $\langle V \rangle$ is more difficult. We present the values of $f_s^{(N)}$ obtained numerically by noticing the limits $\langle V \rangle \to 0$, and $T_{\text{coll}} \to \infty$, in Table S1 — they match quite well.

Table. S1. Comparison of values of stall forces obtained numerically by monitoring the limits $\langle V \rangle \to 0$, and $T_{\text{coll}} \to \infty$. ATP/GTP Concentrations are taken to be $c = 1\mu M$ for actin and $c = 100\mu M$ for microtubule (for other parameters see Table 1).

		$f_s^{(1)}$ (pN)	$f_s^{(2)}$ (pN)	$f_s^{(3)}$ (pN)	$f_s^{(4)}$ (pN)
Actin	$\langle V \rangle$ measurement	3.134	6.389	9.619	12.834
	T_{coll} measurement	3.134	6.390	9.616	12.832
MT	$\langle V \rangle$ measurement	16.748	35.010	52.793	70.384
	T_{coll} measurement	16.741	35.017	52.814	70.473

Appendix C: Results for $\delta = 0.8$

In the main text, we considered the force distribution factor $\delta = 1$ (i.e. only the polymerisation rate is force dependent), as estimated from the experiments on microtubules [7], and consistent with the earlier theories [2,3,5]. This estimation of δ for microtubule has been debated [8]. For actin the value of δ is not known. To check if our results hold for $\delta \neq 1$ (i.e. when both the polymerisation and depolymerisation rates are force dependent) we calculate different dynamical quantities with $\delta = 0.8$ for actin filaments — this is shown in Fig. S2. We see in Fig. S2a that the average cap sizes for filament number $N > 1$ are orders of magnitudes higher than $N = 1$ at large forces — this is similar to the $\delta = 1$ case (although the cap sizes for $N > 1$ do not saturate with force). In Fig. S2b we show the diffusion constant D for the length fluctuations versus force for $\delta = 0.8$ — this figure is quite similar to its counterpart for $\delta = 1$ (see Fig. 8a in main paper). The diffusion constant D does not decay with force for $N > 1$ unlike the $N = 1$ case. Finally in Fig. S2c, we show the diffusion constant D_s derived from the fluctuations of switching events between the capped and uncapped states as a function of force. The figure shows qualitatively similar behavior as the length diffusion constant D. This again demonstrates our point (already shown

for $\delta = 1$) that the overall length fluctuations are closely related to the fluctuations of the switching events between the capped and uncapped states. Thus our conclusions seem to remain qualitatively unchanged even for $\delta \neq 1$.

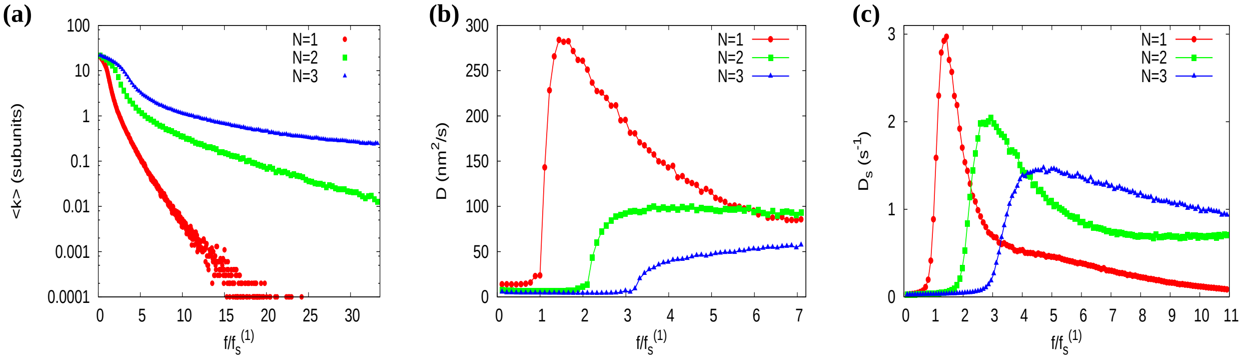

Fig. S2. (a) Average cap size $\langle k \rangle$, (b) the diffusion constant D for the system-length fluctuations, and (c) the diffusion constant D_s for the fluctuations of switching events between capped and uncapped states — these are plotted against the scaled force $f/f_s^{(1)}$. All data are for actin parameters (see Table 1) with a concentration $c = 0.2\mu M$ and for $\delta = 0.8$.

References

1. Dogterom M, Leibler S (1993) Physical aspects of the growth and regulation of microtubule structures. Phys Rev Lett 70: 1347–1350.

2. Padinhateeri R, Kolomeisky AB, Lacoste D (2012) Random Hydrolysis Controls the Dynamic Instability of Microtubules. Biophys J 102: 1274-1283.

3. Tsekouras K, Lacoste D, Mallick K, Joanny JF (2011) Condensation of actin filaments pushing against a barrier. New J Phys 13: 103032.

4. Dipjyoti D, Dibyendu D, Ranjith P (2014) Collective force generated by multiple biofilaments can exceed the sum of forces due to individual ones. New J Phys 16: 063032.

5. Ranjith P, Lacoste D, Mallick K, Joanny JF (2009) Nonequilibrium Self-Assembly of a Filament Coupled to ATP/GTP Hydrolysis. Biophys J 96: 2146-2159.

6. van Doorn GS, Tanase C, Mulder BM, Dogterom M (2000) On the stall force for growing microtubules. Eur Biophys J 20: 2-6.

7. Dogterom M, Yurke B (1997) Measurement of the Force-Velocity Relation for Growing Microtubules. Science 278: 856-860.

8. Kolomeisky AB, Fisher ME (2001) Force-velocity relation for growing microtubules. Biophys J 80: 149 -154.
